# Text Messaging Versus Postal Reminders to Improve Participation in a Colorectal Cancer Screening Program: Randomized Controlled Trial

**DOI:** 10.2196/64243

**Published:** 2025-01-01

**Authors:** Nuria Vives, Gemma Binefa, Noemie Travier, Albert Farre, Jon Aritz Panera, Berta Casas, Carmen Vidal, Gemma Ibáñez-Sanz, Montse Garcia, M-TICS research group

**Affiliations:** 1 Catalan Institut of Oncology Hospitalet del Llobregat Spain; 2 Early Detection of Cancer Research Group Epidemiology, Public Health, Cancer Prevention and Palliative Cures Program Bellvitge Biomedical Research Institute Hospitalet del Llobregat Spain; 3 Consortium for Biomedical Research in Epidemiology and Public Health (CIBEResp) Madrid Spain; 4 School of Health Sciences University of Dundee Dundee United Kingdom; 5 Gastroenterology Department Bellvitge University Hospital Hospitalet del Llobregat Spain; 6 Colorectal Cancer Group Molecular Mechanisms and Experimental Therapeutics in Oncology Program Bellvitge Biomedical Research Institute Hospitalet del Llobregat Spain; 7 see Acknowledgments

**Keywords:** text message, mobile health, colorectal cancer, screening, participation, reminders, text messaging, colorectal cancer screening, fecal immunochemical test

## Abstract

**Background:**

Mobile phone SMS text message reminders have shown moderate effects in improving participation rates in ongoing colorectal cancer screening programs.

**Objective:**

This study aimed to assess the effectiveness of SMS text messages as a replacement for routine postal reminders in a fecal immunochemical test–based colorectal cancer screening program in Catalonia, Spain.

**Methods:**

We conducted a randomized controlled trial among individuals aged 50 to 69 years who were invited to screening but had not completed their fecal immunochemical test within 6 weeks. The intervention group (n=12,167) received an SMS text message reminder, while the control group (n=12,221) followed the standard procedure of receiving a reminder letter. The primary outcome was participation within 18 weeks of the invitation. The trial was stopped early, and a recovery strategy was implemented for nonparticipants in the intervention group. We performed a final analysis to evaluate the impact of the recovery strategy on the main outcome of the trial. Participation was assessed using a logistic regression model adjusting for potential confounders (sex, age, and deprivation score index) globally and by screening behavior.

**Results:**

The trial was discontinued early in September 2022 due to the results of the interim analysis. The interim analysis included 5570 individuals who had completed 18 weeks of follow-up (intention-to-treat). The SMS text message group had a participation rate of 17.2% (477/2781), whereas the control group had a participation rate of 21.9% (610/2789; odds ratio 0.71, 95% CI 0.62-0.82; *P*<.001). As a recovery strategy, 7591 (72.7%) out of 10,442 nonparticipants in the SMS text message group had an open screening episode and received a second reminder by letter, reaching a participation rate of 23% (1748/7591). The final analysis (N=24,388) showed a participation rate of 29.3% (3561/12,167) in the intervention group, which received 2 reminders, while the participation rate was 26.5% (3235/12,221) in the control group (odds ratio 1.16, 95% CI 1.09-1.23; *P*<.001).

**Conclusions:**

Replacing SMS text messages with reminder letters did not increase the participation rate but also led to a decline in participation among nonparticipants 6 weeks after the invitation. However, sending a second reminder by letter significantly increased participation rates among nonparticipants within 6 weeks in the SMS text message group compared with those who received 1 postal reminder (control group). Additional research is essential to determine the best timing and frequency of reminders to boost participation without being intrusive in their choice of participation.

**Trial Registration:**

ClinicalTrials.gov NCT04343950; https://www.clinicaltrials.gov/study/NCT04343950

## Introduction

Colorectal cancer (CRC) is a significant global health concern, with millions of new cases diagnosed and thousands of lives lost each year [1,2]. Early detection through screening is vital for reducing the morbidity and mortality associated with CRC [3,4]. However, despite the proven benefits of early detection, participation rates in CRC screening programs remain suboptimal in most parts of the world [5]. A recent systematic review that analyzed the effectiveness of invitation schemes in fecal occult blood test (FOBT)–based CRC screening found that reminders had a positive impact, increasing participation by 8.5% to 15.8% points depending on the method used [6]. The current European guidelines for CRC screening recommend using reminders through evidence-based communication channels such as letters and telephone reminders to improve cancer screening rates; however, some of these approaches can be costly [4]. Therefore, in order to broaden the reach of screening efforts, there is a need to explore and establish innovative and cost-effective strategies aimed at improving screening uptake among the target population.

In the contemporary era of digital communication, mobile phones have become indispensable tools that can influence various aspects of health care delivery. SMS text messaging has emerged as a versatile and convenient channel for health care professionals to connect with patients. Its utility lies in its accessibility, affordability, and effectiveness as a platform for conveying health-related information, reminders, and motivation [7-9]. SMS text messaging has also shown promising results in addressing some challenges presented by CRC screening programs to overcome certain traditional barriers. A study conducted in Israel found that sending SMS reminders increased participation by 1.8% points compared with no reminder [10]. However, a study in the United Kingdom showed that adding an SMS reminder to the standard letter reminder resulted in an increase in participation only among first-time invitees [11]. In addition, a systematic review of SMS text message interventions for cancer screening found that they had a small effect on CRC screening rates, ranging from 0.6% to 3.3% [12]. There is, therefore, a need for further research to explore the potential of this approach more comprehensively.

This randomized controlled trial (RCT) assessed the effectiveness of SMS text messages versus postal reminders in a fecal immunochemical test (FIT)–based CRC screening program and is part of the larger Mobile Phone Messaging as a Tool to Improve Cancer Screening (M-TICS) study, which aims to evaluate the impact of different SMS text messaging interventions on participation in the ongoing colorectal and breast cancer screening programs in the metropolitan area of Barcelona, Catalonia, Spain [13].

## Methods

### Study Design

We conducted an RCT that compared an SMS text message reminder with the standard reminder procedure (a letter sent by postal mail) in a FIT-based CRC screening program. Recruitment was conducted from May 9, 2022, to September 9, 2022. The trial was stopped early, and we launched a recovery strategy for nonparticipants in the intervention group.

### Ethical Considerations

The study received ethical approval from the Ethics Committee of the Bellvitge University Hospital (reference PR042/20), which waived the requirement to obtain the participant’s signature as part of the consent process, as the intervention was a minor variation on the invitation practice. The study was performed under Good Clinical Practice and the Declaration of Helsinki. The data utilized in this study were de-identified prior to analysis to ensure the protection of individuals' privacy. The participants in this study did not receive any form of compensation.

### Setting

The Catalan Institute of Oncology screening hub, as part of the CRC screening program in Catalonia, Spain, manages a biennial FIT-based program for CRC in northern and southern metropolitan areas of Barcelona and covers a target population of 502,348 men and women aged 50-69 years (January 1, 2022). The hub identifies individuals due for screening from the Central Register of Insured Persons of the Catalan Health Service. Eligible individuals receive an invitation letter to pick up a FIT kit at any pharmacy participating in the CRC program. A reminder invitation letter is sent to those nonparticipants in the sixth week. A total of 10 weeks after the reminder letter, the screening episode is automatically closed as a nonparticipant if no response is received. Community pharmacies collect and send completed FIT kits to their allocated laboratory to be processed. Individuals with positive FIT results are offered a diagnostic colonoscopy. If an invitation letter is returned to our hub due to an incorrect mailing address, we verify its current and correct status. If the address is wrong, we send a new letter to the updated address.

### Participants and Randomization

Eligible individuals were men and women who received an invitation letter but did not participate within 6 weeks. Simple randomization was performed to allocate the participants. We designed an application using JavaScript’s built-in *Math.random* function to select and randomize eligible individuals in a 1:1 ratio stratified by previous screening behavior to either the intervention or the control group. From May 9, 2022, onward, eligible individuals were randomized to the intervention weekly until the target sample size was achieved. Individuals without a registered mobile phone were excluded. No blinding was considered at any step. However, the end point of this study did not require subjective judgment.

### Intervention Description

Individuals randomly assigned to the control group received the standard reminder procedure: a reminder letter 6 weeks after the invitation. Individuals randomly assigned to the intervention group received an SMS text message reminder 6 weeks after the invitation instead of the reminder letter. SMS text messages were bidirectional (enabling 2-way messaging) and fully automated delivery through a platform. The screening hub staff members managed the incoming individual responses. The initial of the first name and the entire last name were part of the SMS text message. A link to request a hard copy of the original invitation letter was provided, as this includes a unique code needed to pick up the FIT kit in their local pharmacy. A reminder letter was sent to individuals whose SMS text messages failed to be delivered (Multimedia Appendix 1).

### Outcomes and Baseline Variables

The primary outcome was the screening participation defined as individuals who completed an adequate FIT kit (positive or negative) within 18 weeks of the invitation. When the laboratory is unable to analyze a test (spoilt kit or technical failure), the participant is asked to repeat the FIT. If they refuse, the final test result is coded as indeterminate. Baseline variables were sex, age at the invitation, previous screening behavior (first-time invitees, previous nonparticipants, and previous screenees), and tertiles of deprivation score (DS) index based on the individual’s Catalan primary health care referral area [14].

### Sample Size

The sample size was estimated to detect differential effects on previous screening behavior (first-time invitees, previous nonparticipants, and previous screenees). We hypothesized that SMS text message reminders would boost the participation of first-time invitees. A small, nonsignificant effect was expected for previous nonparticipants or previous screenees.

We considered a proportion difference in participation of 3% points between the intervention and control groups for each previous screening stratum.

Participation rates after 6 weeks from the invitation letter of 9%, 17%, and 52% were considered for first-time invitees, previous nonparticipants, and previous screenees (based on our retrospective data). We anticipated that 10% of the phone numbers would be wrongly recorded. Finally, accepting an α risk of .05 and a power of 0.90 in a 2-tailed test, the total sample size was estimated at 25,572 individuals.

### Interim Analysis

We performed an interim analysis when about 25% of the individuals recruited had completed the 18-week follow-up period. The criterion we used to stop the trial early was based on the Haybittle-Peto boundary, which sets a threshold of *P*<.001 when 1 interim analysis is performed while maintaining the α level and statistical power determined in the sample size calculation [15].

### Recovery Strategy

A recovery strategy was designed to be implemented if results from interim analyses met the criteria for stopping the intervention. The recovery strategy involved a postal reminder for all nonrespondents within the intervention group if they were still eligible to participate (SMS text message reminder received within the last 10 weeks). This strategy aimed to reduce nonparticipation in the intervention group and, therefore, mitigate the impact of the nonresponse of the intervention group and prevent ethical issues.

### Statistical Analysis

All primary analyses were conducted as an intention-to-treat analysis, including all eligible individuals, regardless of whether they received the complete or partial intended intervention. We compared baseline characteristics between the study groups using the 2-tailed Student *t* test for continuous data and chi-square tests for categorical data to identify imbalances during randomization. Similarly, we made comparisons of baseline characteristics in trial groups based on previous screening behavior. The effectiveness of the SMS text message reminders in participation was assessed using a logistic regression model adjusting for potential confounders (sex, age, and DS index) globally and by screening behavior. Results were reported as odds ratio (OR) and 95% CIs. A 2-sided *P* value of less than .05 was considered statistically significant. We also performed a post hoc subgroup analysis according to sex, age, and DS index. For the post hoc comparisons, we applied the Bonferroni correction by setting a significance level of *P*<.03 when 2 tests were conducted (for each sex and each age group) and a threshold of *P*<.02 when 3 tests were performed (one for each DS index). All the analyses were performed using Stata (version 18.0; StataCorp LP).

## Results

### Participants

**Figure 1:**
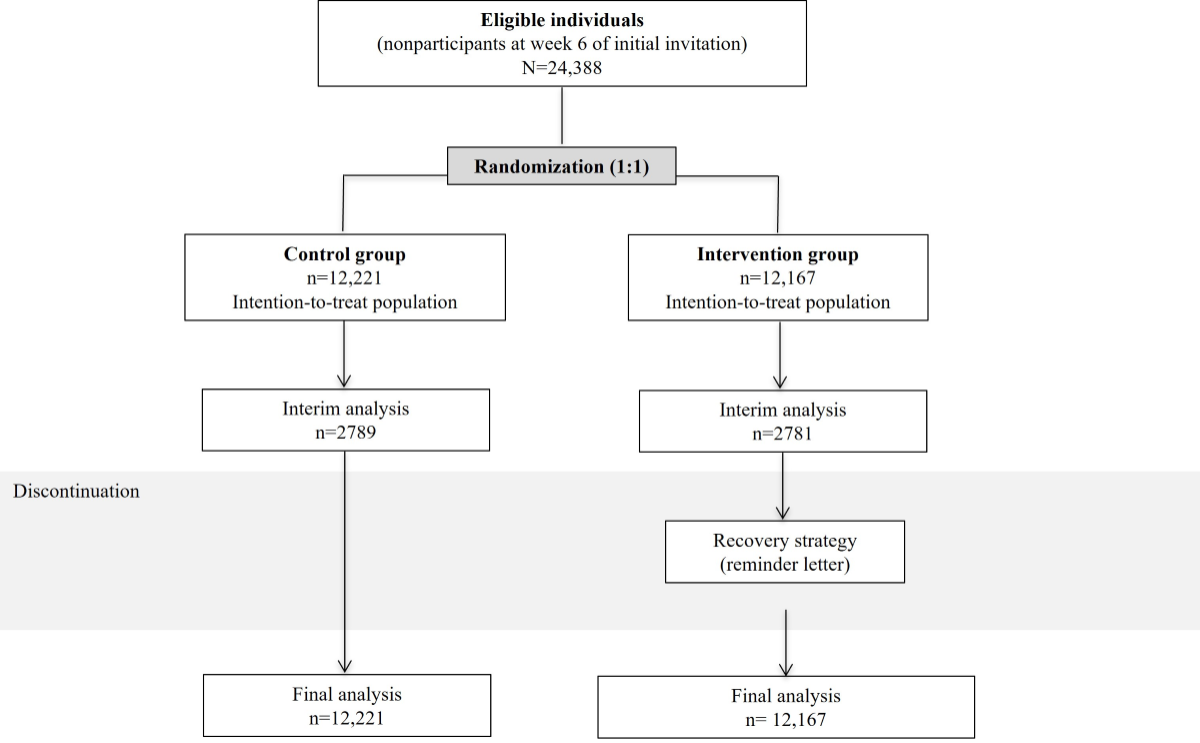
CONSORT (Consolidated Standards of Reporting Trials) flow diagram of reminder intervention to participate in a colorectal cancer screening program.

Between May 9 and September 9, 2022, a total of 24,388 individuals were enrolled in this study. A total of 12,167 individuals were randomly assigned to the SMS text message group and 12,221 to the control group (Figure 1). SMS text messages failed to be delivered to 457 (3.8%) out of 12,167 individuals in the intervention group, and letters failed to be delivered to 625 (5.1%) out of 12,221 individuals in the control group. Furthermore, a total of 113 individuals who were lost follow-up due to changes in their individual addresses were classified as nonparticipants. The intention-to-treat approach included all individuals selected and randomized, regardless of their final status.

### Interim Analysis

The interim analysis included a sample of 5570 individuals who had completed the 18 weeks of follow-up, thus representing 23% of the total 24,388 individuals recruited. Among these 5570 individuals, 2825 (50.7%) were women, the mean age was 57.1 (SD 5.78) years, 2123 (38.1%) were from the high DS area, and 2613 (46.9%) had previously undergone a screening test. Baseline characteristics were similar in both groups (Multimedia Appendix 2). The participation rate of nonparticipants within 6 weeks of invitation was significantly higher in the letter group (610/2789, 21.9%) compared with the SMS text message group (477/2781, 17.2%) at 18 weeks, with an OR of 0.71 (95% CI 0.62-0.82). The stratified analysis by previous screening behavior also showed differences in participation between the two groups, favoring the letter group (Table 1).

**Table 1:** Interim analysis of the participation by previous screening behavior among nonparticipants within 6 weeks of invitation (intention-to-treat).

Previous screening behavior	Letter, n/N (%)	SMS text message, n/N (%)	OR^a^ (95% CI)	*P* value
Previous screenees	475/1315 (36.1)	400/1298 (30.8)	0.79 (0.67-0.92)	.004
First-time invitees	135/1474 (9.2)	77/1483 (5.2)	0.55 (0.4-0.76)	<.001
Previous nonparticipants	32/651 (4.9)	11/585 (1.9)	0.38 (0.19-0.76)	.004
Global	610/2789 (21.9)	477/2781 (17.2)	0.71 (0.62-0.82)	<.001

^a^OR: odds ratio.

### Recovery Strategy

**Figure 2:**
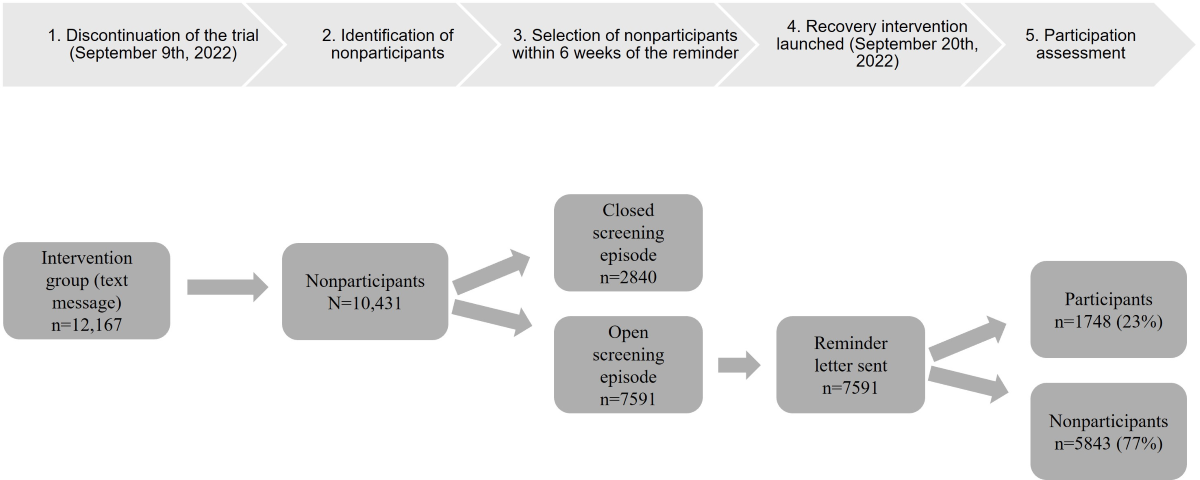
Diagram of the recovery strategy launched in the intervention group among individuals with an open screening episode. If no response is received 6 weeks after the reminder letter, the screening episode is automatically closed as a nonparticipant.

At the time of the trial interruption, we identified 10,431 individuals who did not participate in the SMS text message group. Of these individuals, 7591 (72.7%) were still eligible to participate as they received a reminder (SMS text message) fewer than 10 weeks ago. Therefore, we sent a second reminder by letter to encourage them to participate and help mitigate the negative impact of the SMS text message (Figure 2). The time of sending reminder letters varied between 11 and 103 days after receiving the SMS text message. The screening participation rate among those who received a second reminder by letter was 23% (1748/7591). However, participation was higher among those to whom we sent the reminder letter fewer than 30 days before (528/1652, 31%) than when the reminder letter was sent more than 60 days after the initial SMS text message reminder (594/3221, 18.4%; Table 2)

**Table 2:** Participation in the intervention group by the time the recovery strategy was launched following the SMS text message reminder.

	Participants, n/N (%)	*P* value
Fewer than or equal to 30 days	528/1652 (32)	<.001
Between 31 and 60 days	626/2718 (23)	<.001
More than 60 days	594/3221 (18.4)	<.001
Global	1748/7591 (23)	—^a^

^a^Not applicable.

**Figure 3:**
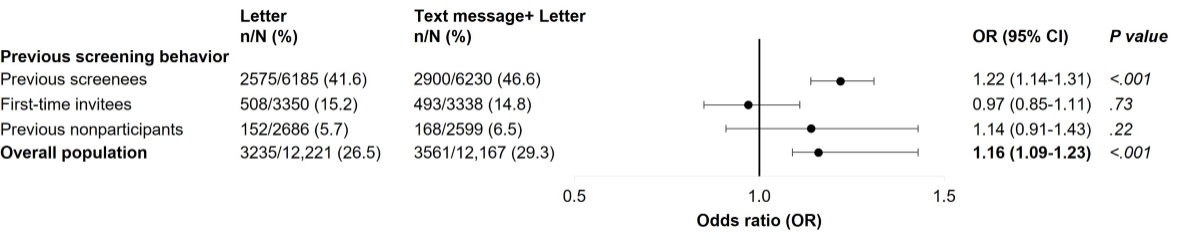
Impact of the recovery strategy on participation globally and by screening behavior (intention-to-treat). The analysis was adjusted for sex, age, and deprivation score, considering the letter group as a reference.

The overall population baseline characteristics distribution was similar in both trials’ groups (Multimedia Appendix 2). When stratified by previous screening behavior, overall population baseline characteristics also remained similar (Multimedia Appendix 3). According to the data presented in Figure 3, the sending of a reminder letter as a recovery strategy for nonparticipants from the intervention group resulted in a significantly higher final screening participation rate of 29.3% (3561/12,167) as compared with the letter group (3235/12,221, 26.5%), with an OR of 1.16 (95% CI 1.09-1.23). The stratified analysis by previous screening behavior showed a higher participation rate in the SMS text message group after the recovery strategy was launched compared with the letter group among previous screenees (OR 1.22, 95% CI 1.14-1.31). No significant differences were observed in participation rates of first-time invitees and previous nonparticipants after sending the reminder letter in the intervention group compared with the letter group (Figure 3).

Post hoc subgroup analyses showed that the recovery strategy of sending a reminder letter to previous nonparticipants in the SMS text message group significantly increased overall final participation, regardless of sex and age group, compared with the control group. However, when compared with the control group, significant increases in participation in the intervention group were only observed in the first and third tertile of the DS index (Multimedia Appendix 4).

## Discussion

### Principal Findings

This 2-arm RCT examined an SMS text message reminder intervention versus the usual reminder letter to nonparticipants in a FIT-based CRC screening program within 6 weeks of the invitation. The interim analysis showed that the participation rate was 4.5% points lower when an SMS text message reminder was sent than the usual reminder letter. This decline in participation in the intervention group was consistent when stratified by previous screening behavior. As a result, the intervention was discontinued, and a recovery strategy was implemented, which involved sending a second reminder by letter to nonparticipants in the intervention group. These combined reminders (SMS text message followed by a letter) led to an increase of 2.8% points in the final participation in the intervention group after the recovery strategy compared with the usual reminder letter group.

Previously published studies have shown variable results regarding the effectiveness of SMS text message reminders for increasing uptake in CRC screening, depending on the invitation scheme used. Our program’s global participation rate is around 45%. Out of the total participants, 70% participated within 6 weeks, while the remaining 30% required a reminder to participate. Therefore, using reminders is crucial to ensure acceptable levels of participation. As far as we know, no studies compared the impact of replacing a letter reminder with an SMS text message reminder. However, a study in the United Kingdom found that adding a second reminder through SMS text message did not improve overall participation compared with a reminder letter. Still, it did show a small benefit for newly screened participants [11]. In contrast, our study showed that replacing a letter with SMS text message reminders had a negative effect on both global participation and on each stratum of previous screening behavior. First-time invitees are crucial population subgroups for boosting participation in cancer screening programs because they are more likely to adhere to regular screening if engaged positively. On the other hand, previous nonparticipants have missed earlier opportunities, and efforts to engage them might require more intensive interventions, such as addressing specific concerns or barriers. Previous screenees have already participated, indicating they understand the importance of screening. Efforts to boost their participation might be less critical since they are likely to continue attending screenings without additional encouragement.

The obtained results were unexpected, as SMS text messaging is currently a routine part of cancer screening programs in the metropolitan Area of Barcelona. For example, SMS text messaging has been used as an appointment reminder for screening mammography since 2019, with an improvement in attendance rates [16]. Also, in other studies of the M-TICS project, we found that an SMS text message reminder to complete the FIT kit increased participation among individuals who had picked up the kit from the pharmacy but did not return it within 14 days [17]. There are different possible explanations for this unexpected result. First, letters (invitations or reminders) from our CRC screening program contain specific codes required for picking up FIT kits from the pharmacy. These codes serve as a unique identification for the recipients and are necessary for acquiring the kits. This requirement may have created a logistical barrier to access screening in the intervention group because the SMS text message alone did not allow them to pick up the test kit [18]. As a result, they had to present the invitation letter to the pharmacy, and if they were unable to locate it, they had to contact the screening hub to request a new invitation letter to be sent to them. Second, pharmacists who collaborated with the CRC screening program were informed by email that SMS text message reminders would be sent as part of a study to improve participation in the program. However, it is possible that not all the pharmacy clerks received the information, or its content was not sufficiently clear instructions about what they should explain to individuals who show up at their pharmacy with an SMS text message to pick up a FIT kit. This lack of dissemination may be partly responsible for the failure of the SMS text message approach. Providing precise and easy-to-follow instructions to all involved parties is essential to ensure better participation rates in similar programs.

In our study, we randomly assigned nonparticipants to receive a single reminder through letter or SMS text message at 6 weeks. When we stopped the intervention and sent a second reminder by letter to those nonparticipants in the SMS text message arm, we finally reached higher participation in the intervention than those in the letter arm, where individuals only received one reminder to participate in our CRC screening program. These findings are of particular importance as they highlight that the use of more than 1 reminder, such as SMS text messaging and postal delivery, can be more effective than using only one. In this regard, 2 previous studies found that the sending of 2 reminders combined with a prenotification message significantly increased participation rates compared with a single reminder [19,20]. We should investigate the potential impact of sending more than one SMS text message reminder in our program, carefully establishing the timing and frequency of reminders to maximize effectiveness without causing annoyance or being intrusive in their choice of participation [21].

### Conclusions

Although our initial attempt to replace a letter with an SMS text message reminder did not achieve the desired result, we eventually found that sending more than 1 reminder combining both SMS text messaging and postal delivery methods may increase participation rates. Integrating SMS text messages into cancer screening should ensure that the screening process is streamlined and minimize access barriers. When implementing an intervention, all stakeholders should be informed and receive clear and concise instructions. In doing so, we will facilitate that more people participate in cancer screening programs and receive the benefits of early detection and timely treatment.

## Appendix

Multimedia Appendix 1 Content of the text message and reminder letter sent to nonparticipants.

Multimedia Appendix 2 Baseline characteristics of enrolled individuals by trial arm (intention-to-treat population).

Multimedia Appendix 3 Baseline characteristics of enrolled individuals by previous screening behavior and trial arm (intention-to-treat population).

Multimedia Appendix 4 Subgroup analysis of the impact of the recovery strategy on participation (intention-to-treat).

Multimedia Appendix 5 CONSORT (Consolidated Standards of Reporting Trials) 2020 checklist.

## Abbreviations

CRC: colorectal cancer
DS: deprivation score
FIT: fecal immunochemical test
FOBT: fecal occult blood test
M-TICS: Mobile Phone Messaging as a Tool to Improve Cancer Screening
OR: odds ratio
RCT: randomized controlled trial
