# Supplementary material for: Text Messaging Versus Postal Reminders to Improve Participation in a Colorectal Cancer Screening Program: Randomized Controlled Trial
Source: JMIR Mhealth Uhealth. 2025 Jan 1;13:e64243. doi: 10.2196/64243 (PMC11736219; doi:10.2196/64243)
Supplement: Multimedia Appendix 1 [file mhealth_v13i1e64243_app1.docx]

**CONTENT OF THE TEXT MESSAGE AND REMINDER LETTER SENT TO NON-PARTICIPANTS SIX WEEKS FROM THE INVITATION**

**text message reminder**

X SURNAME. ICO reminds you to collect your FIT kit for colorectal cancer prevention at the PHARMACY. Your code XXXXXXX. Follow instructions: link


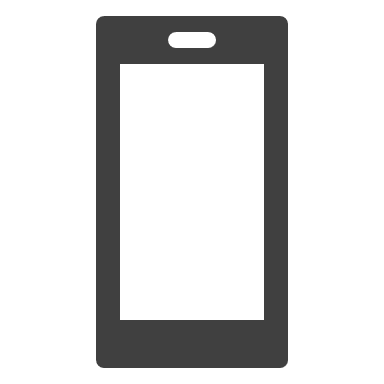


**The link instructions:**

*You can pick up the screening test at any PHARMACY in your residential area displaying these badges:*


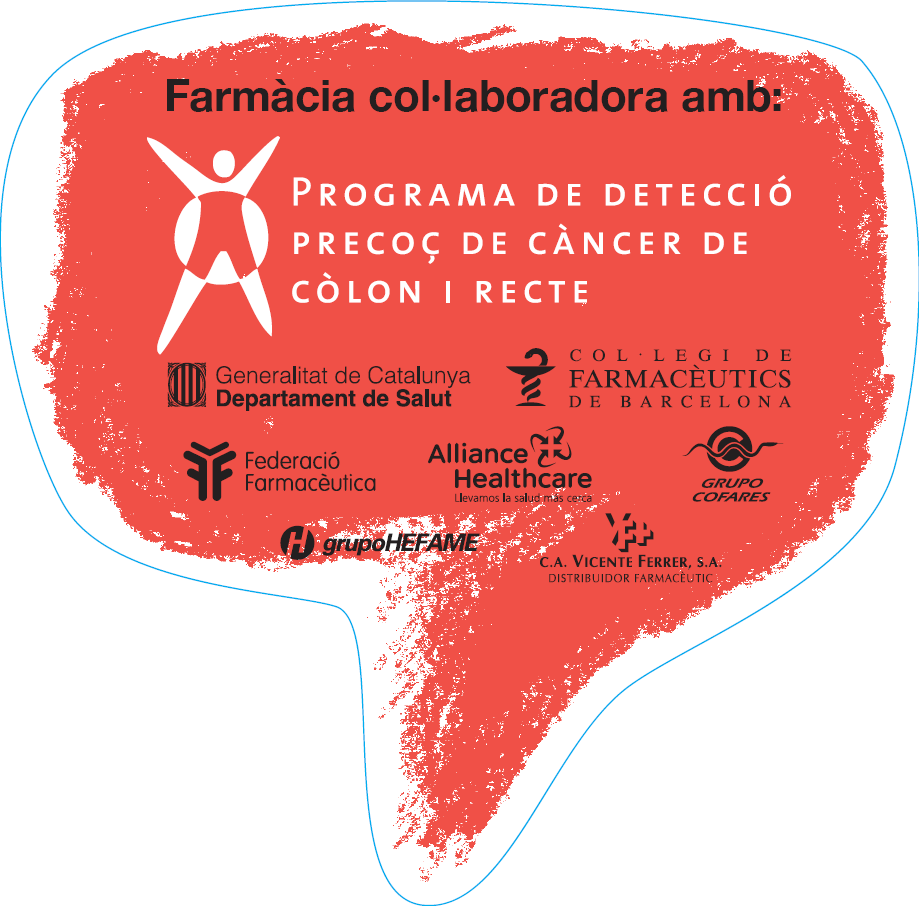


***IT IS ESSENTIAL THAT YOU CARRY THE LETTER*** ***OF THE PROGRAM WE SENT YOU A FEW DAYS AGO.***

*Please, contact us if you have any questions.*

***Colorectal Cancer Screening Program***

***Telephone and email***

***Horari:*** *de dilluns a divendres, de 9 del matí a 3 de la tarda*

Name of the Coordinator of the Colorectal Cancer Screening Program

**STANDARD REMINDER LETTER**

Dear,

A few weeks ago, we invited you to participate in the **Colorectal Cancer Screening Program**. The program consists of performing a simple test **every two years, free of charge**. The test consists of collecting a small sample of stool to search for blood that cannot be detected with the naked eye.

You have **one month** from the date of this letter to pick up the test at one of the pharmacies (list attached**). IT IS ESSENTIAL THAT YOU CARRY THIS LETTER** so that they can provide you with the test kit. Once you have the test done, you must return it to the pharmacy as soon as possible. In a few weeks, you will receive the result by letter and/or telephone.

If you have been previously diagnosed with colorectal cancer, ulcerative colitis, or Crohn's disease, please contact us before going to the pharmacy.

| 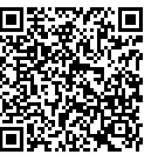 **Colorectal Cancer Screening Program**  **Telephone**  **Email**  **Web:** <https://www.icoprevencio.cat/colon/cat/>  **Office hours** |
| --- |

**Prevention is ALSO in your hands**

Sincerely,

Name of the Coordinator of the Colorectal Cancer Screening Program

The Catalan Institute of Oncology, in compliance with Regulation (EU) 2016/679 of the European Parliament and of the Council of 27 April 2016 on the protection of individuals with regard to the processing of personal data and on the free movement of such data, informs you that the processing of your data will be to ensure the registration, management, and monitoring of the information arising from your participation in the program, as well as for health research purposes always in an anonymized form.

This information will be used by the administrative services and services directly linked to the health care of our entity, each in its competencies, and may be sent in whole or in part to public and private official bodies which, for legal reasons or for reasons of material necessity, must have access to the data for the purposes of the correct provision of the medical-health care that constitutes the purpose of the processing of these data.

The data provided will be kept in accordance with the health legislation in force at any given time. You have the right to exercise your rights of access, rectification, deletion, limitation of processing, portability, and opposition of your data by writing to the Data Protection Officer at lopd@iconcologia.net, in any case, you must attach a photocopy of your national identity card or equivalent.

Likewise, you are informed of your right to file a complaint at any time before the Catalan Data Protection Authority if you do not agree with the treatment carried out by our entity or consider your rights violated.
