# Supplementary material for: Text Messaging Versus Postal Reminders to Improve Participation in a Colorectal Cancer Screening Program: Randomized Controlled Trial
Source: JMIR Mhealth Uhealth. 2025 Jan 1;13:e64243. doi: 10.2196/64243 (PMC11736219; doi:10.2196/64243)
Supplement: Multimedia Appendix 2 [file mhealth_v13i1e64243_app2.docx]

**Multimedia Appendix 2. Baseline characteristics of enrolled individuals by trial arm (intention to treat population)**

|  | **Interim population**  **(23% of enrollees)** | | |  |  | **Overall population**  **(100% of enrollees)** | | |  |
| --- | --- | --- | --- | --- | --- | --- | --- | --- | --- |
|  | **Text message** | | **Letter** |  |  | **Text message** | | **Letter** |  |
|  | **n (%)** |  | **n (%)** | ***P value*** |  | **n (%)** |  | **n (%)** | ***P value*** |
| **Sex** |  |  |  |  |  |  |  |  |  |
| Men | 1,351 (48.6) |  | 1,394 (50.0) | *0.295* |  | 5,922 (48.7) |  | 5,932 (48.5) | *0.835* |
| Women | 1,430 (51.4) |  | 1,395 (50.0) |  |  | 6,245 (51.3) |  | 6,289 (51.5) |  |
| **Age, years** |  |  |  |  |  |  |  |  |  |
| Mean (SD) | 56.9 (5.79) |  | 57.3 (5.84) | *0.040* |  | 57.0 (5.78) |  | 57.2 (5.79) | *0.905* |
| **Age groups, years** |  |  |  |  |  |  |  |  |  |
| 50-59 | 1,921 (69.0) |  | 1,878 (67.3) | *0.163* |  | 8,253 (67.8) |  | 8,299 (67.9) | *0.898* |
| 60-69 | 860 (31.0) |  | 911 (32.7) |  |  | 3,914 (32.2) |  | 3,922 (32.1) |  |
| **Deprivation Score** |  |  |  |  |  |  |  |  |  |
| 1st tertile | 512 (18.4) |  | 529 (18.9) | *0.601* |  | 3,827 (31.4) |  | 3,835 (31.4) | *0.581* |
| 2nd tertile | 1,191 (42.8) |  | 1,215 (43.6) |  |  | 2,625 (21.6) |  | 2,702 (22.1) |  |
| 3rd tertile | 1,078 (38.8) |  | 1,045 (37.5) |  |  | 5,715 (47.0) |  | 5,684 (46.5) |  |
| **Previous screening behavior** | |  |  |  |  |  |  |  |  |
| Previous screenees | 1,298 (46.7) |  | 1,315 (47.2) | *0.032* |  | 6,230 (51.2) |  | 6,185 (50.6) |  |
| First-time invitees | 898 (32.3) |  | 823 (29.5) |  |  | 3,338 (27.4) |  | 3,350 (27.4) | *0.396* |
| Non-participants | 585 (21.0) |  | 651 (23.3) |  |  | 2,599 (21.4) |  | 2,686 (22.0) |  |
| **Total** | **2,781** |  | **2,789** |  |  | **12,167** |  | **12,221** |  |
