# Supplementary material for: Text Messaging Versus Postal Reminders to Improve Participation in a Colorectal Cancer Screening Program: Randomized Controlled Trial
Source: JMIR Mhealth Uhealth. 2025 Jan 1;13:e64243. doi: 10.2196/64243 (PMC11736219; doi:10.2196/64243)
Supplement: Multimedia Appendix 3 [file mhealth_v13i1e64243_app3.docx]

**Multimedia Appendix 3. Baseline characteristics of enrolled individuals by previous screening behavior and trial arm (intention to treat population)**

|  |  | **New for screening** | | |  |  |  | **Previous participants** | | |  |  |  | **Non-previous participants** | | |  |
| --- | --- | --- | --- | --- | --- | --- | --- | --- | --- | --- | --- | --- | --- | --- | --- | --- | --- |
|  |  | **Text-message** | | **Letter** |  |  |  | **Text-message** | | **Letter** |  |  |  | **Text-message** | | **Letter** |  |
|  |  | **n (%)** |  | **n (%)** |  | ***P value*** |  | **n (%)** |  | **n (%)** |  | ***P value*** |  | **n (%)** |  | **n (%)** | ***P value*** |
| Sex |  |  |  |  |  |  |  |  |  |  |  |  |  |  |  |  |  |
| Men |  | 1,774 (53.1) |  | 1,717 (51.2) |  | *0.121* |  | 2,835 (45.5) |  | 2,801 (45.3) |  | *0.807* |  | 1,313 (50.5) |  | 1,414 (52.6) | *0.122* |
| Women |  | 1,564 (46.9) |  | 1,633 (48.8) |  |  |  | 3,395 (54.5) |  | 3,384 (54.7) |  |  |  | 1,286 (49.5) |  | 1,272 (47.4) |  |
| Age, years |  |  |  |  |  |  |  |  |  |  |  |  |  |  |  |  |  |
| Mean (SD) |  | 51.3 (2.80) |  | 51.4 (3.06) |  | *0.151* |  | 59.5 (4.98) |  | 59.4 (5.00) |  | *0.253* |  | 59.2 (5.04) |  | 59.3 (5.04) | *0.284* |
| Age groups, years |  |  |  |  |  |  |  |  |  |  |  |  |  |  |  |  |  |
| 50-59 |  | 3,231 (96.8) |  | 3,222 (96.2) |  | *0.172* |  | 3,482 (55.9) |  | 3,543 (57.3) |  | *0.117* |  | 1,540 (59.2) |  | 1,534 (57.1) | *0.114* |
| 60-69 |  | 107 (3.2) |  | 128 (3.8) |  |  |  | 2,748 (44.1) |  | 2,642 (42.7) |  |  |  | 1,059 (40.8) |  | 1,152 (42.9) |  |
| Deprivation Score |  |  |  |  |  |  |  |  |  |  |  |  |  |  |  |  |  |
| 1st tertile |  | 963 (28.9) |  | 983 (29.3) |  | *0.743* |  | 2,083 (33.4) |  | 2,072 (33.5) |  | *0.956* |  | 781 (30.1) |  | 780 (29.0) | *0.413* |
| 2nd tertile |  | 873 (26.1) |  | 891 (26.6) |  |  |  | 1,206 (19.4) |  | 1,208 (19.5) |  |  |  | 546 (21.0) |  | 603 (22.5) |  |
| 3rd tertile |  | 1,502 (45.0) |  | 1,476 (44.1) |  |  |  | 2,941 (47.2) |  | 2,905 (47.0) |  |  |  | 1,272 (48.9) |  | 1,303 (48.5) |  |
| **Total** |  | **3,338** |  | **3,350** |  |  |  | **6,230** |  | **6,185** |  |  |  | **2,599** |  | **2,686** |  |
