# Supplementary material for: Text Messaging Versus Postal Reminders to Improve Participation in a Colorectal Cancer Screening Program: Randomized Controlled Trial
Source: JMIR Mhealth Uhealth. 2025 Jan 1;13:e64243. doi: 10.2196/64243 (PMC11736219; doi:10.2196/64243)
Supplement: Multimedia Appendix 4 [file mhealth_v13i1e64243_app4.docx]

**Multimedia Appendix 4. Subgroup analysis of the impact of the recovery strategy on participation (intention-to-treat)**

|  |  | **Overall population (n= 24,388)** | | |  |  |  |  |
| --- | --- | --- | --- | --- | --- | --- | --- | --- |
|  |  | Letter* n/N (%) |  | Text message + recovery strategy n/N (%) |  | OR (95% CI) |  | *P value* |
| **Sex** |  |  |  |  |  |  |  |  |
| Women |  | 1,765 /6,289 (28.1) |  | 1,935/ 6,245 (31) |  | 1.17 (1.08-1.27) | | *<0.001* |
| Men |  | 1,470 /5,932 (24.8) |  | 1,626/ 5,922 (27.5) |  | 1.15 (1.05-1.26) | | *0.002* |
| **Age** |  |  |  |  |  |  |  |  |
| 50-59 |  | 2,071/ 8,299 (25) |  | 2,186/ 8,253 (26.5) |  | 1.11 (1.03-1.2) |  | *0.024* |
| 60-69 |  | 1,164/ 8,253 (29.7) |  | 1,375 /3,914 (35.1) |  | 1.26 (1.14-1.4) |  | *<0.001* |
| **Deprivation Score** |  |  |  |  |  |  |  |  |
| 1st tertile |  | 1,145/ 3,835 (29.9) |  | 1,264 /3,827 (33) |  | 1.18 (1.06-1.31) | | *0.003* |
| 2nd tertile |  | 653 /2,702 (24.2) |  | 665 /2,625 (25.3) |  | 1.04 (0.91-1.19) | | *0.324* |
| 3rd tertile |  | 1,437/ 5,684 (25.3) |  | 1,632/ 5,715 (28.6) |  | 1.21 (1.1-1.32) |  | *<0.001* |
| **Total** |  | **3,235 (26.5)** |  | **3,561/ 12,167 (29.3)** |  | **1.16 (1.09-1.23)** | | *<0.001* |

**Reference group (adjusted for sex, age, Deprivation Score, and previous screening behavior)*
